# The Aix-La-Chapelle Treatment of Syphilis
1Journal of Balneology and Climatology, July, 1904.


**Published:** 1904-08-20

**Authors:** 


### THE AIX-LA-CHAPELLE TREATMENT OF SYPHILIS.[1]

The thermal springs of Aix-la-Chapelle are no more able than any other medicinal waters to cure syphilis. Their value depends on the fact that they facilitate the use of mercury—the only real specific competent to eradicate the virus and to exercise a favourable influence at all stages of the disease. It is particularly desirable to concentrate as much treatment as possible into the first few years, or even months, for only in this way can the development of the tertiary stage be avoided. Mercurial inunction is to be preferred to administration *per os* as producing a more energetic result and being free from the risk of intestinal complications. It is this method which is almost exclusively employed at Aix, and it is claimed to have more permanent effects than those obtained through other channels of administration. To secure satisfactory absorption of the remedy it is necessary that the skin be properly prepared. This is accomplished by means of the thermal baths, which soften the epidermis and dilate the orifices of the glands. The inunction is performed by a certificated "frotteur," who massages the prescribed quantity of the ointment into the skin. It is believed that the mercury is distributed through the body as an albuminate; and as douche-massages, thermal, vapour, and electric baths increase the amount excreted in the urine, these are employed with a view to quicken the metabolic processes, in which of course mercury plays its appropriate part. To avoid any risk of stomatitis, disinfection of the mouth with dentifrices and mouth-washes is practised. Every morning the patient drinks two to three glasses of the spring-water, to which, if there is sluggishness of the bowels, a teaspoonful of Aix-la-Chapelle salts is added. This is followed by a thermal bath. The portion of the surface that is to be the site of the next rubbing is then thoroughly cleansed with soap and water, and care is taken to avoid the use of soap or of friction by the towel over parts which have previously been subject to inunction. Occasionally a vapour bath or douche-massage precedes the ordinary thermal bath. Next the patient is instructed to rest in bed, and about an hour after breakfast the inunction is performed. The ointment used is a 33 per cent. grey ointment, and this is applied in different parts according to a regular daily sequence—legs, thighs, back, abdomen and iliac regions, arms. Before the midday meal another glass of spring water is taken with a view to promote appetite. After every meal the patient uses a salol and chlorate of potassium tooth-paste, and in addition he employs every half-hour a solution of aluminium acetico-tartaricum as a mouth-wash. The diet during the whole of the treatment should be as supporting as possible. Milk should be taken freely. Red wine with seltzer-water may be permitted with meals. Smoking, especially during the secondary period, is apt to provoke mucous plaques on the mouth and throat. Frequent observation is made of the urine, and the development of albuminuria demands a pause in the treatment.

The above are the details of an ordinary course of inunction. In particular circumstances supplementary measures may be necessary. Thus any signs of gummatous development call for potassium iodide, which is given in doses of 75 to 100 grains dissolved in milk or soda-water. When the gumma subsides iodide is no longer necessary, but to be on the safe side injections of iodipin (a 25 per cent. solution of iodine in sesamoid oil) may be given. This insures a protracted action, as it is found that even six months after the injection of 200 grains of iodipin the urine still gives a definite iodine reaction. Iodipin further appears to increase the tolerance for the mercurial inunction, and this is continued simultaneously. Another supplementary remedy is sarsaparilla. This is indicated where former excessive mercurial treatment has so influenced the tissues that a prompt response to the inunction is not obtained. It also has a favourable influence on recurring secondary and tertiary affections of the buccal mucous membrane.

In exceptional instances where, in consequence of an extensive scaly eruption or of undue sensitiveness of the skin, inunction cannot be practised, hypodermic injection of salicylate of mercury in oil emulsion may be ordered twice weekly, the other features of the treatment as already described being continued.

Dr. Lieven is opposed to the commencement of treatment before the secondary stage is developed. The first course lasts about six weeks. Three subsequent courses are taken at intervals of six months. If at the end of a year after the third course no relapse should occur, the next course may be postponed for another 12 months. After this the cure is generally complete.

The article of which the above paragraphs contain an abstract is exceptionally complete in detail, and will well repay perusal by any one interested in the subject.

[1] Journal of Balneology and Climatology, July, 1904.

